# Potential anti-tumor effects of *Solenopsis invicta* venom

**DOI:** 10.3389/fimmu.2023.1200659

**Published:** 2023-05-22

**Authors:** Yizhang Mo, Qingxing Shi, Guojun Qi, Kebing Chen

**Affiliations:** ^1^ Department of Spine Surgery, The Sixth Affiliated Hospital, Sun Yat-sen University, Guangzhou, China; ^2^ Guangdong Provincial Key Laboratory of High Technology for Plant Protection, Plant Protection Research Institute, Guangdong Academy of Agricultural Science, Guangzhou, Guangdong, China

**Keywords:** *Solenopsis invicta* Buren, venom, alkaloid, tumor, antitumor activity, Solenopsin A

## 1 Introduction

Stinging by social Hymenoptera species such as honeybees, vespids, and ants is a major causes of anaphylaxis (1). Hymenopteran stings only cause minor local inflammation and reactions in most people. However, patients with venom allergy are at risk for systemic allergic reactions, which are a leading cause of anaphylaxis fatalities (2). Fire ants (*Solenopsis*) are aggressive species and named for the burning pain they inflict. Indeed, stinging ant venom allergy has become a significant public health concern in parts of the world where the fire ants are endemic (3).

Red imported fire ant (RIFA), *Solenopsis invicta* Buren, is a major notorious invasive ant species that appears in the list of 100 of the world’s worst invasive alien species (4). It can inflict serious economic and ecological damage on households, electricity service, communications, wildlife, agriculture, recreation areas, and bring a huge threat to human health and life (5, 6). Given the widespread distribution of RIFA in human-inhabited areas, reports of fire ant attacks and stings are common. For instance, more than 30% of people in fire ant-infested areas have suffered stings each year in the southeast region of the United States and China (6, 7).

RIFA is of major medical importance, and its toxicity mechanism is fairly unique. RIFA venom mainly including water-insoluble alkaloid differs from the venoms of honeybees, vespids, which are composed largely of protein-containing aqueous solutions (2, 8). Undoubtedly, it is the venom that makes RIFA such a significant health hazard to humans. However, RIFA venom also has a positive side which can significantly inhibit some key symptoms of psoriasis and malaria (9). Therefore, exploring the biological role of RIFA venom and making them beneficial will provide a new scheme for the development and application of RIFA. Here, we summarize the current research on RIFA venoms and put forward the idea that RIFA venoms have potential anti-tumor effects.

## 2 Components of red imported fire ant venom and its physiological activity

After being stung by RIFA, human body will feel burning pain. A few people will have allergic reactions to toxic proteins, and even allergic shock in severe cases (1, 10, 11). The powerful virulence of S. invicta is closely related to its venom gland secretions, which are produced in the poison gland and mainly composed of insoluble alkaloids and trace protein (2, 8, 12, 13). Four protein antigens (SoliI-IV, allergic proteins) were identified in water-soluble protein peptides (small molecules and enzymes) of the fire ant venom, which can cause allergic reactions (14–17). The insoluble alkaloids are mainly composed of 2-methyl-6-alkyl or alkenyl piperidines and piperideines, which can promote mast cells to release histamine and vasoactive amines, causing cell necrosis, pain and abscess (18, 19). Alkaloids with many kinds of activities were identified from the RIFA venom. These small nitrogen heterocyclic compounds have a variety of pharmacological activities. 2-methyl-6-undecylpiperidine (solenopsin A) is a powerful poison hemolysin and skin necrosis of alkaloids that make the cells release histamine (19, 20). cis- and trans-2-methyl-6-undecylpiperidine (isosolenopsin A and solenopsin A) interferes with the coupling between ion channels and the recognition sites of vertebrate nicotinic acetylcholine receptors (21). After intravenous injection of the alkaloids into mice, it was found that the alkaloids can seriously damage the central nervous system and cardiovascular system of mice, indicating that the alkaloids can penetrate the blood-brain barrier and cause dizziness, seizures, cardiopulmonary complications, death and other consequences when the injection dose ranges from 3-30 mg/kg (21). Solenopsin A and analogs can ceramide and help to restore the barrier function of the skin (9, 22). Experiments have proved that some key symptoms of psoriasis can be significantly inhibited. When the local area of the lesion treated with solenopsin A was studied by immunohistochemistry, it was found that the number of CD4+ T cells, CD8+ T cells and CD11c+ dendritic cells decreased significantly. RIFA venom may become a new therapeutic method for the development of psoriasis treatment (22, 23). In addition, solenopsins can inhibit the formation of *Pseudomonas fluorescens* and other biofilms, and reduce bacterial adhesion significantly (24).

Collectively, RIFA venom has a variety of active ingredients, and plays a variety of biological functions, and can even regulate human physiological functions. Therefore, RIFA venom has the potential to be converted into clinical therapeutic drugs.

## 3 Biological venom and tumor treatment

Malignant tumor is one of the most serious diseases threatening human health. Surprisingly, a variety of biological venoms, such as bee venom, snake venom, toad venom and scorpion venom, have been found to have therapeutic effects on tumor (25–32). These venoms promote apoptosis, autophagy and lysis of tumor cells by regulating gene expression of tumor cells and cytotoxicity. Inhibition of tumor cell proliferation, adhesion, migration and invasion, inhibition of tumor angiogenesis and other effects to play an anti-tumor role (25–32). These biotoxins are mainly composed of peptides, proteins and alkaloids. It is worth noting that bee venom, snake venom, toad venom and scorpion venom can promote tumor cell apoptosis by inhibiting the activation of phosphatidylinoinosiol 3-kinase (PI3K) and phospho-Akt (p-Akt). And inhibit tumor angiogenesis to shrink tumor volume (25–32). Interestingly, solenopsin A in RIFA venom inhibited the angiogenesis of zebrafish by delaying the formation of angiogenesis precursor or bud *in vivo* (33). *In vitro* and cellular experiments, solenopsin A showed relatively selective inhibition of Akt activation in a competitive manner with ATP. In addition, in cellular experiments, solenopsin A also regulated the downstream pathway by inhibiting PI3K activation (33). The PI3K/Akt pathway is involved in the regulation of various cellular functions *in vivo*, including proliferation, cytoskeletal organization, survival, and carcinogenesis (34–38). Akt is an important drug target for cancer and inflammatory diseases. Akt inhibitors are divided into ATP competitive Akt inhibitors and allosteric Akt inhibitors. At present, the effectiveness and specificity of Akt inhibitors are not satisfactory, and have a variety of adverse reactions (39). Therefore, the development of tumor therapeutics targeting Akt has a broad application prospect. It is worth noting that the active components in the above biological venom, such as bee venom, toad venom, snake venom and scorpion venom, can not only regulate PI3K/Akt pathway, but also inhibit angiogenesis through Akt/VEGF pathway and mTOR/VEGF pathway, inhibit tumor growth by regulating the expression levels of cyclin, p21, p27, p38 and HIF- 1 α pathway and promote tumor cell lysis and apoptosis by up-regulating the expression of RIP1, RIP3, PARP-1 and ERK signaling pathway (29, 40–42). Therefore, the anti-tumor effect of RIFA venom through other pathways remains to be further explored. In addition, as mentioned earlier, RIFA venom plays a role in regulating the number of immune cells in psoriasis, in which CD4+T cells and CD8+T cells play an important role in anti-tumor immunity. Therefore, RIFA venom may play an anti-tumor effect by regulating the immune system (22, 43, 44).

**Figure 1:**
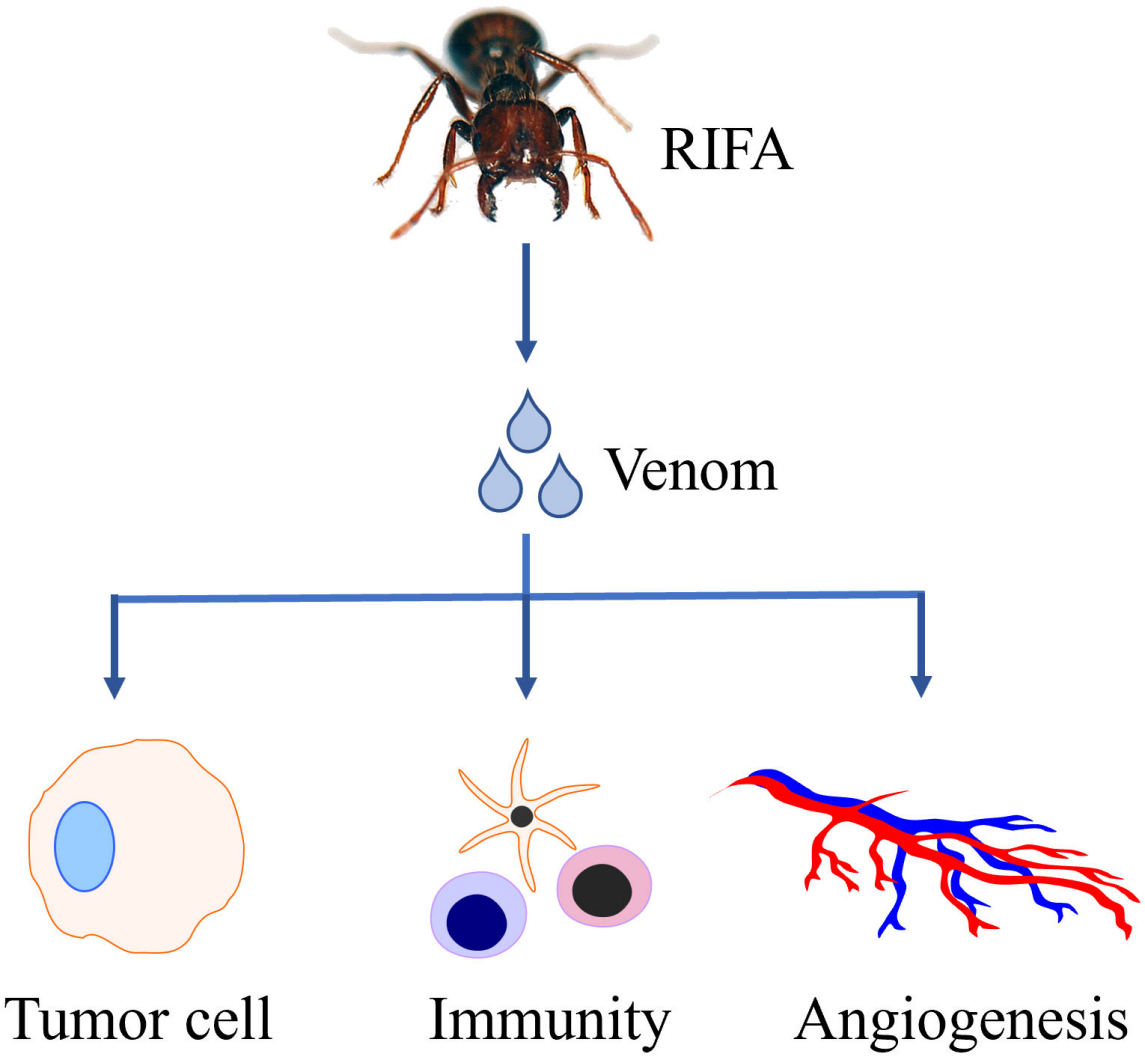
The venom secreted by red imported fire ant (RIFA) has potential anti-tumor effect.

Collectively, RIFA venom is likely to exert its anti-tumor effect through PI3K pathway, regulation of angiogenesis, regulation of immune system and other unknown pathways (**Figure 1**).

## 4 Discussion

The invasion of RIFA has caused serious economic losses and ecological disasters (45, 46). After being stung of the fire ant, people will have pain, allergic reactions, and even allergic shock (1, 10, 11). RIFA venom is mainly composed of allergic proteins (SoliI-IV) and alkaloids that can cause allergic reactions (14, 15). To explore the biological effects of RIFA venom will be helpful for the potential clinical therapeutic agent for tumor treatment. Many physiological effects of alkaloid components in RIFA venom have been explored, such as promoting mast cells to release histamines and vasoactive amines, causing cell necrosis, causing pain and pustular reaction at the sting site (18, 19), promoting hemolysis and skin necrosis (19), interfering with the coupling between ion channels and the recognition sites of vertebrate nicotinic acetylcholine receptors. It can cause damage to the central nervous system and cardiovascular system (21), promote the recovery of skin barrier function (9, 22), regulates the number of local immune cells in the lesion (23), etc. It is worth noting that solenopsin can inhibit Akt and inhibit angiogenesis *in vitro* through PI3K signaling pathway (33). These findings are consistent with the fact that biotoxins such as bee venom, snake venom, toad venom, and scorpion venom can promote tumor cell apoptosis and reduce tumor size by inhibiting tumor angiogenesis by inhibiting the activation of phosphatidylinostat 3-kinase (PI3K) and phospho-Akt (p-Akt) (25–32). Bee venom, snake venom, toad venom and scorpion venom have been found to play an anti-tumor role by regulating gene expression of tumor cells, cytotoxicity, promoting apoptosis, autophagy and lysis of tumor cells, inhibiting tumor cell proliferation, adhesion, migration and invasion, and inhibiting tumor angiogenesis. However, there are few studies on RIFA venom, and only the PI3K/Akt pathway has been found to play its anti-tumor function, the PI3K/Akt pathway is closely related to malignant tumors, and Akt is an important drug target for cancer and inflammatory diseases. Therefore, the development of Akt targeted tumor drugs has a wide application prospect. In addition, RIFA venom can also regulate the number of immune cells, and its anti-tumor effect may be exerted by regulating the immune system, either. Finally, many components of RIFA venom need to be further explored and their valuable functions and applications explored. Unfortunately, the evolutionary reason for anti-tumor function of ant venom is currently unknown based on limited evidence. We speculate that interactions might exist between ants and mammals, shaping the physiology for both animals. The technology of separation and synthesis of active components in RIFA venom has been maturing, which makes it possible for us to study and utilize specific venom (14, 47).

Collectively, RIFA venom has a variety of effects and has the potential to be used as a clinical therapeutic agent. Its inhibitory effect on P13K-Akt pathway is similar to that of bee venom, snake venom, toad venom and scorpion venom, suggesting that RIFA venom has potential anti-tumor ability.

## Author contributions

Conceptualization, data curation, writing-original draft preparation, writing-review and editing: all authors. Supervision and funding acquisition: KC, GQ. All authors have read and agreed to the final version of the manuscript.
